# Development and Validation of a Questionnaire to Measure Digital Maturity of General Practitioner Practices: Web-Based Cross-Sectional Survey Study

**DOI:** 10.2196/81416

**Published:** 2025-10-14

**Authors:** Timo Neunaber, Achim Mortsiefer, Sven Meister

**Affiliations:** 1 Health Care Informatics, Faculty of Health School of Medicine Witten/Herdecke University Witten Germany; 2 General Practice II and Patient-Centeredness in Primary Care, Institute of General Practice and Primary Care Faculty of Health, School of Medicine Witten/Herdecke University Witten Germany; 3 Department of Healthcare Fraunhofer Institute for Software and Systems Engineering Dortmund Germany

**Keywords:** digital health, eHealth, digital maturity, maturity assessment, maturity model, primary care, general practitioner, GP, GP practice, online survey, questionnaire, validation

## Abstract

**Background:**

The digital transformation in health care requires valid instruments to assess the level of digitalization. Maturity models are widely used to measure the digital maturity level of institutions. To date, however, there is no standardized and empirically grounded measurement tool for general practices in outpatient care.

**Objective:**

This study aimed to identify and validate the key dimensions of digital maturity in order to develop a questionnaire to measure the digital maturity of general practices. The development of a questionnaire was intended to advance research into maturity models in outpatient care and provide general practitioners (GPs) and policymakers with a tool for the digital transformation process.

**Methods:**

A web-based cross-sectional survey study was conducted among GPs in Germany. Based on exploratory factor analysis (EFA), the underlying dimensions of digital maturity were first identified. The factor structure was then examined using confirmatory factor analysis (CFA). After evaluating convergent and discriminant validity, the overall model fit was assessed using fit indices. Following model adjustments based on modification indices, the final questionnaire was established. Finally, we calculated the digital maturity level for our sample, both for each individual dimension and as an overall score, by computing the mean value for each dimension and an overall mean across all dimensions.

**Results:**

Responses from 201 GPs were included in the data analysis. We identified and validated 6 dimensions of digital maturity, comprising 16 items. Both convergent and discriminant validity were confirmed. The model fit was excellent (robust comparative fit index [CFI]=0.993; robust Tucker-Lewis index [TLI]=0.990; robust root mean square error of approximation [RMSEA]=0.022; *P* value of close fit [PCLOSE]=.98; standardized root mean square residual [SRMR]=0.043). The questionnaire included six dimensions: effects of digitalization, participation of practice staff, maturity of the practice management system, staff competencies and sense of responsibility, IT security and data protection, and digitally supported processes. The scale showed good internal consistency (overall Cronbach α=.809). In our sample, the overall digital maturity averaged 3.77 out of 5, with the highest maturity observed in IT security and data protection (mean 4.45, SD 0.61) and the lowest in effects of digitalization (mean 3.1, SD 1.0).

**Conclusions:**

This is the first study in which the dimensions of digital maturity in outpatient care for GP practices have been empirically identified and validated as the basis for developing a questionnaire. The findings provide a foundation for further research on measuring digital maturity in outpatient care and for advancing the development of digital maturity models. The questionnaire allows GPs to assess their practices’ digitalization, identify areas for improvement (eg, infrastructure, staff skills), and support internal strategy or benchmarking. For policymakers, it offers a standardized tool to plan support measures and monitor digitalization progress.

## Introduction

### Background

Factors such as demographic change, the shortage of specialists, and the uneven distribution of general practitioner (GP) practices between rural and urban areas mean that it is often no longer possible to ensure locally available and easily accessible care for patients. However, GPs are often the first point of contact for patients and, in many health care systems, also serve as gatekeepers who coordinate further medical treatment [1]. The use of digital solutions offers a promising opportunity to effectively address both current and future challenges in outpatient care [2]. GPs can implement digital apps in situations in which practices face limited resources, such as staffing shortages, or where patients are required to travel long distances. Examples include electronic patient records; telemedicine services, such as video consultations or telemonitoring; and health apps. Administrative processes in GP practices can also be streamlined through intelligent appointment scheduling systems. Overall, digitalization is believed to have the potential to contribute to more efficient and effective health care delivery [3,4].

Maturity models are increasingly being used to strategically advance digital transformation and to manage various initiatives. For example, in its global strategy on digital health, the World Health Organization emphasizes the importance of assessing the digital maturity of health care systems to support national funding decisions [5]. Maturity models were therefore developed “to assess the maturity (i.e. competency, capability, level of sophistication) of a selected domain based on a more or less comprehensive set of criteria” [6]. Standardized questionnaires serve to measure digital maturity, reflecting the current state of digitalization in organizations such as GP practices. The results can be used to evaluate one’s own level of digitalization, benchmark against other practices, and even identify specific areas for investment [7-9].

So far, however, the understanding of digital maturity in GP practices remains fragmented. Research on digital maturity, its dimensions, and the development of maturity models is still at an early stage [10]. There are only initial efforts to measure the level of digital maturity in general practice care, such as the measurement model developed by the Westphalia-Lippe Association of Statutory Health Insurance Physicians in Germany [11,12] and the Gippsland Primary Health Network model in Australia [13]. Widely used measurement instruments, such as questionnaires for assessing digital maturity in general practices, do not currently exist. This stands in stark contrast to inpatient care, where the Electronic Medical Record Adoption Model (EMRAM), developed by the Healthcare Information and Management Systems Society (HIMSS), serves as a widely adopted international maturity model [14]. In Germany, the Hospital Future Act (KHZG) established DigitalRadar as a national instrument for measuring the digital maturity of hospital [15,16]. Moreover, research on the measurement dimensions of digital maturity is significantly more advanced [17].

To address the lack of maturity models in GP care, scientifically validated measurement instruments, based on psychometric testing, are needed. This requires the empirical validation of both dimensions and items, a step that is often criticized as missing in the development of maturity models [18]. Currently, however, such validation is absent from the few existing scientific publications on measuring digital maturity in GP practices [19-22]. Instead, frameworks or dimensions from other areas of health care are often applied.

Against this background, the authors took an initial step in foundational research by identifying 6 dimensions and 26 subcategories of digital maturity for GP practices as part of a qualitative research project [23].

### Objective

The aim of this study was to validate the dimensions of digital maturity and develop a corresponding instrument to measure the level of digitalization in GP practices. The research question guiding our work was, “Which dimensions of digital maturity in GP practices can be empirically identified and validated to develop a reliable and valid measurement instrument?”

The overarching goal of this study was to advance the research and development of maturity models in outpatient care in order to assess the digital transformation of GP practices and support them throughout the transformation process. Our study built directly on our previous research on the digital maturity of GP practices [10,23].

## Methods

### Study Design

To answer the research question, we conducted a cross-sectional online survey among GPs in Germany. The survey design followed the CHERRIES (Checklist for Reporting Results of Internet E-Surveys) [24]; see Multimedia Appendix 1 for the completed CHERRIES and Multimedia Appendix 2 for the questionnaire in English and German. The survey was open and administered using the free online survey platform LimeSurvey [25]. Participants were therefore drawn from a random sample.

### Ethical Considerations

Ethical approval was granted by the Ethics Committee of Witten/Herdecke University (application number S-47/2023). Participants were required to provide informed consent by agreeing to the data protection policy before taking part in the survey. They were also informed about all relevant aspects of the study, including the survey duration, data storage period and location, the researchers responsible, and the purpose of the study.

### Structure of the Questionnaire

The section of the questionnaire relevant to this study consisted of 6 pages and included a total of 43 items. The number of items per page ranged from 3 to 15. Completion took approximately 10-15 minutes. If participants did not fully complete a page, the survey application displayed a notification. Participants also had the option to review and change their responses using the Back button. Where applicable, questions were randomized within the survey. Adaptive questioning was not used.

At the beginning of the survey, demographic characteristics—including gender, age, type of practice, and population size of the practice location—were collected. In addition, participants were asked to assess the average digital health literacy of their patients on a scale from 0 to 5 and the digitalization level of their practice on a scale from 0 to 10. This was followed by 37 items covering 5 dimensions of digital maturity. In total, data was collected on 37 items on these dimensions using a 5-point Likert scale from 1 (strongly disagree) to 5 (strongly agree). The data collected from these items served as the basis for identifying and validating the dimensions and development of the questionnaire.

### Item Construction and Pretest

The starting point was an item repository developed based on findings from previous expert interviews on the dimensions of digital maturity in GP practices [23]. These dimensions included “digitally supported processes,” “skills and competencies of the practice team,” “organizational structures and rules,” “technical infrastructure,” and “effects of digitalization.” In the absence of existing measurement instruments, either new items were developed or items were adopted from comparable studies. For instance, items related to the “skills and competencies of the practice team” dimension were adapted from the German TAEG questionnaire, which measures technological affinity [26]. Similarly, items in the “technical infrastructure” dimension were informed by the System Usability Scale [27]. Item development was carried out by an expert panel in collaboration with representatives of the medical profession and medical interest groups in order to incorporate both theoretical and practice-oriented perspectives.

Subsequently, a pretest was conducted in accordance with the recommendations of Schnell et al [28], involving 20 participants. Half of the participants were GPs, while the other half consisted of specialists and experts in the field of digital health with extensive professional experience. The pretest followed the “frame of reference probing” methodology [28]. Based on the pretest results, we drew initial conclusions regarding content validity, reliability, and item difficulty and distribution. After the pretest was completed, revisions were made to the introductory text, the individual questions, and the response options. The final items and the abbreviations used can be found in Multimedia Appendix 3.

### Recruiting

Participant recruitment was conducted between October 2024 and January 2025. Participants were recruited through multiple channels. The majority were contacted via GP associations, institutes of general practice, general practice research networks, teaching practices, and mailing lists directed at GP practices. Additionally, GPs were approached through Associations of Statutory Health Insurance Physicians, medical chambers, physician networks, and personal contacts. Third, social media platforms, such as LinkedIn (Microsoft), were also used. Finally, the snowball sampling method was used: on the final page of the survey, participants were encouraged to share the survey with other eligible colleagues from their networks. We typically contacted multipliers—such as physician networks—initially via email or over the phone. These individuals then shared the survey link through internal communication channels and announcements. The survey was promoted primarily through online means, including emails, newsletters, and websites. Participation was voluntary, and no incentives were provided by the authors.

### Data Cleaning

The data were cleaned using liter recommendations [29,30] with the help of SPSS software (IBM Corp) [31]. The stepwise data-cleaning process is presented in Table 1.

**Table 1:** Stepwise data cleaning process with exclusion criteria and results.

Exclusion criterion	Result after data cleaning	Value, n (%)
—^a^	Clicked the survey link and consented to the data policy	375 (100.0)
No items completed	Participated in the survey (at least one item completed)	321 (85.6)
>1 item page not completed	Participated with at least one fully completed item page	230 (61.3)
Further missing responses	Fully completed the survey	212 (56.5)
Careless response behavior	Careful response behavior confirmed	202 (53.9)
Illogical response behavior	Logical response behavior confirmed	201 (53.6)
Violation of ≥5-fold k-anonymity	The k-anonymity requirements met	201 (53.6)

^a^Not applicable.

Due to our settings in the LimeSurvey app, data were collected only if participants clicked the survey link and provided informed consent by agreeing to the data protection policy (N=375). In the first step, we systematically checked the data sets for completeness. A total of 230 (61.3%) participants completed at least one item page. After excluding 18 (4.8%) participants due to isolated missing values, 212 (56.5%) participants remained with fully completed data sets. Given the minimal loss of information, we decided not to apply any imputation procedures. In the second step, we excluded data sets that indicated careless or illogical response behavior (n=11, 2.9%). Finally, we checked compliance with k-anonymity requirements. Data sets with fewer than five values in the demographic variables were excluded. In total, of the 375 individuals who accessed the survey, 201 (53.6%) responses were included in the final data analysis.

### Data Analysis

First, we conducted exploratory factor analysis (EFA) using SPSS [31] to identify the latent dimensions of digital maturity. In the second step, we evaluated the factor-analytical model using confirmatory factor analysis (CFA), performed with R version 4.4.3 (R Foundation for Statistical Computing) [32] and the *lavaan* add-on package [33].

For EFA, we followed the guidelines of Watkins [34] and the recommendations of Samuels [35]. We began by testing the data for normality using the Kolmogorov-Smirnov test. Since normality could not be confirmed, we chose principal axis factoring as the estimation method, in line with Fabrigar et al [36]. We also applied oblique (promax) rotation, as correlations between the underlying dimensions were expected [37]. The number of factors was determined using parallel analysis [38]. Items with low communalities, low factor loadings, or high cross-loadings were removed. Once the factor model was stabilized, we assessed the total variance explained by the retained factors, the sampling adequacy using the Kaiser-Meyer-Olkin (KMO) measure and Bartlett’s test of sphericity, as well as the average extracted communality of the variables.

For CFA, we followed the reporting framework proposed by Gaskin et al [39]. Due to the nonnormal distribution of the data, we used the robust maximum likelihood (MLR) estimation method. To assess validity, we examined both discriminant and convergent validity at the factor level. Discriminant validity was evaluated using the Fornell-Larcker criterion [40] and the recommendations of Rönkkö and Cho [41]. For convergent validity, we considered the average variance extracted (AVE) [42].

At the model level, we assessed the model fit using the robust comparative fit index (CFI), the robust Tucker-Lewis index (TLI), the robust root mean square error of approximation (RMSEA), the *P* value of close fit (PCLOSE), and the standardized root mean square residual (SRMR), given the small sample size and the nonnormality of the data. Modification indices were also analyzed to identify opportunities for improving the model fit. After finalizing the questionnaire, we evaluated the reliability of the scale as a whole and for each dimension using the Cronbach α coefficient [43]. Next, we calculated the digital maturity level for our sample, both for each individual dimension and as an overall score. For this purpose, we computed the mean value for each dimension as well as an overall mean across all dimensions.

## Results

### Demographic Characteristics

The demographic characteristics of the study participants are summarized in Table 2.

**Table 2:** Demographic characteristics of surveyed GPs^a^ and their practices (N=201).

Characteristics		Value
**Sex, n (%)**		
	Male	124 (61.7)
	Female	77 (38.3)
**Age (years), n (%)**		
	20-40	22 (10.9)
	41-60	134 (66.7)
	>60	45 (22.4)
**Type of practice, n (%)**		
	Solo practice	92 (45.8)
	Shared practice	24 (11.9)
	Group practice	66 (32.8)
	Medical care center	19 (9.5)
**Practice location (population size), n (%)**		
	<5000 inhabitants	42 (20.9)
	5000-20,000 inhabitants	57 (28.4)
	20,001-100,000 inhabitants	43 (21.4)
	100,001-500,000 inhabitants	32 (15.9)
	>500,000 inhabitants	27 (13.4)
Digitalization level of GP practice (scale from 0 to 10), mean (SD)		7.11 (1.71)
Average digital health literacy of patients (scale from 0 to 5), mean (SD)		2.57 (0.95)

^a^GP: general practitioner.

Of the 201 participants included in the analysis, the majority of GPs were male, aged between 41 and 60 years, and primarily worked in solo practices. Most GP practices were located in small towns with populations between 5000 and 20,000 inhabitants. On average, participants rated the digital health literacy of their patients at 2.57 out of 5 (SD 0.95) and the digitalization level of their own practice at 7.11 out of 10 (SD 1.71).

### Explorative Factor Analysis

The results of the EFA are presented in Table 3.

**Table 3:** Results of EFA^a^: factors, items, and loadings.

	Factors and items	Factor loading^b^						
		1	2	3	4	5	6	7
**Factor 1: effects of digitalization**								
	In my medical practice, digitalization has a positive effect on the practice’s business results. (EFF04)	0.923	—^c^	—	—	—	—	—
	In my medical practice, digitalization has a positive impact on the quality of patient care. (EFF01)	0.899	—	—	—	—	—	—
	In my medical practice, digitalization has a positive effect on the workload of the practice staff. (EFF03)	0.791	—	—	—	—	—	—
	In my medical practice, digitalization has a positive effect on patient satisfaction. (EFF02)	0.667	—	—	—	—	—	—
**Factor 2: practice culture**								
	In my medical practice, digital apps are consistently used whenever the situation allows for it. (PC02)	—	0.780	—	—	—	—	—
	In my medical practice, the use of digital apps is standardized. (KQM03)	—	0.628	—	—	—	—	—
	In my medical practice, the use of digital apps is an integral part of daily routines. (PC03)	—	0.612	—	—	—	—	—
	In my medical practice, the use of digital apps is part of our mission statement. (PC01)	—	0.534	—	—	—	—	—
**Factor 3: participation of practice staff**								
	Important decisions regarding digitalization projects are made jointly in our medical practice. (PP03)	—	—	0.897	—	—	—	—
	In my medical practice, the practice owners make decisions about digitalization projects without involving the practice staff. (PP02)^d^	—	—	0.708	—	—	—	—
	When decisions about digitalization projects are pending, the practice staff has sufficient opportunity to contribute. (PP01)	—	—	0.684	—	—	—	—
**Factor 4: maturity of the practice management system**								
	In my medical practice, the practice management system is reliable and stable. (TM03)	—	—	—	0.875	—	—	—
	In my medical practice, the practice management system is easy to use. (TM02)	—	—	—	0.786	—	—	—
	In my medical practice, the practice management system is up to date. (TM01)	—	—	—	0.548	—	—	—
**Factor 5: staff competencies and sense of responsibility**								
	In my medical practice, the team finds it easy to learn how to use digital apps. (TA02)	—	—	—	—	0.814	—	—
	In my medical practice, the staff often feels overwhelmed by new demands related to digitalization. (CM03)^d^	—	—	—	—	0.722	—	—
	In my medical practice, there is often a sense that no one feels responsible for digitalization projects. (RS02)^d^	—	—	—	—	0.534	—	—
**Factor 6: IT security and data protection**								
	In my medical practice, measures are taken to meet IT security requirements. (IDS01)	—	—	—	—	—	0.753	—
	In my medical practice, measures are taken to meet data protection requirements. (IDS02)	—	—	—	—	—	0.753	—
	In my medical practice, employees receive training on how to use IT systems securely when handling patient data. (IDS03)	—	—	—	—	—	0.461	—
**Factor 7: digitally supported processes**								
	In my medical practice, administrative processes (eg, finance, human resources, procurement, internal communication) are digitally supported. (DP02)	—	—	—	—	—	—	0.961
	In my medical practice, core processes (eg, appointment management, patient intake, medical history, diagnostics, treatment, documentation) are digitally supported. (DP01)	—	—	—	—	—	—	0.523
	In my medical practice, communication with external service providers and institutions (eg, specialists, laboratories, health insurance providers) is digitally supported. (DP03)	—	—	—	—	—	—	0.420

^a^EFA: exploratory factor analysis.

^b^The items were translated from German into English. The values of the factor loadings apply to the German items.

^c^Not applicable.

^d^The data for the items PP02, CM03, and RS02 were inverted.

After six iterations of the EFA, seven factors were identified: (1) effects of digitalization, (2) practice culture, (3) participation of practice staff, (4) maturity of the practice management system, (5) staff competencies and sense of responsibility, (6) IT security and data protection, and (7) digitally supported processes. The labels for factors 2 and 5 were newly developed as they comprised cross-thematic items (eg, factor 5 with items TA02, CM03_Invers, RS02_Invers) and therefore differed from the original categorization derived from the expert interviews [23]. A total of 23 variables were assigned to the 7 factors. During the adjustment process, two initial items were removed due to communalities below 0.2, and one item was excluded due to a factor loading below 0.3. Additional items were eliminated due to high cross-loadings or low factor loadings. Both Bartlett’s test of sphericity (*χ*²_253_=2036.241, *P*<.001) and the KMO measure of sampling adequacy (KMO=0.842) indicated that the sample was suitable for factor analysis. The average extracted communality of the items was 0.557, in line with the recommendations of MacCallum et al [44]. The seven factors explained 70.103% of the total variance.

### Confirmatory Factor Analysis

The results for convergent and discriminant validity are presented in Table 4.

**Table 4:** Convergent and discriminant validity demonstrated using AVE^a^, √AVE, and the highest correlation with other factors.

Factor	AVE	Convergent validity met?	√AVE	Highest correlation with another factor	Discriminant validity met?
Factor 1: effects of digitalization	0.698	Yes	0.835	0.640 (with factor 2)	Yes
Factor 2: practice culture	0.493	No	0.702	0.651 (with factor 7)	Yes (with limitations)
Factor 3: participation of practice staff	0.565	Yes	0.752	0.375 (with factor 5)	Yes
Factor 4: maturity of the practice management system	0.548	Yes	0.740	0.270 (with factor 5)	Yes
Factor 5: staff competencies and sense of responsibility	0.515	Yes	0.717	0.603 (with factor 2)	Yes
Factor 6: IT security and data protection	0.467	No	0.683	0.470 (with factor 7)	Yes
Factor 7: digitally supported processes	0.485	No	0.696	0.651 (with factor 2)	Yes (with limitations)

^a^AVE: average variance extracted.

Factors 2 (AVE=0.493), 6 (AVE=0.467), and 7 (AVE=0.485) did not meet the criteria for convergent validity. The discriminant validity analysis revealed a high degree of overlap between factors 2 and 7. In addition, the highest observed correlation between any two factors was 0.8074, which exceeds the recommended threshold of 0.80 proposed by Rönkkö and Cho [41].

Despite these limitations in convergent and discriminant validity, the overall model quality was good. All model fit indices indicated acceptable values (CFI=0.945, TLI=0.933, RMSEA=0.049, PCLOSE=0.523, SRMR=0.063).

Due to the unmet requirements for convergent and discriminant validity, the model was subsequently adjusted.

### Model Adjustment

Items KQM03 (factor 2; loading=0.619), IDS03 (factor 6; loading=0.575), and DP03 (factor 7; loading=0.531) had the lowest factor loadings, indicating potential for improving convergent validity. Table 5 presents the 10 highest modification indices identified in the measurement model.

**Table 5:** Ten highest modification indices identified for the measurement model.

lhs^a^	op^b^	rhs^c^	mi^d^	epc^e^	sepc.lv^f^	sepc.all^g^
Factor3 =~	DP03	16.63	0.370	0.269	0.294	0.294
Factor7 =~	IDS03	13.05	0.538	0.338	0.312	0.312
Factor5 =~	EFF03	12.70	0.351	0.251	0.215	0.215
KQM03 ~~	CM03_Invers	11.80	–0.183	–0.183	–0.297	–0.297
Factor4 =~	DP03	11.01	0.557	0.215	0.236	0.236
Factor5 =~	DP03	10.53	0.383	0.274	0.300	0.300
Factor2 =~	IDS03	10.48	0.305	0.282	0.260	0.260
DP01 ~~	DP03	9.34	–0.156	–0.156	–0.268	–0.268
Factor2 =~	PP02_Invers	9.16	–0.273	–0.252	–0.209	–0.209
EFF03~~	CM03_Invers	8.51	0.123	0.123	0.263	0.263

^a^lhs: left-hand side.

^b^op: operator.

^c^rhs: right-hand side.

^d^mi: modification index.

^e^epc: expected parameter change.

^f^sepc.lv: standardized epc on a latent variable scale.

^g^sepc.all: standardized epc (all).

The modification indices primarily indicated additional cross-loadings, for example, for the items DP03, IDS03, and EFF03. Based on these findings and the results of low factor loadings, the items EFF03, KQM03, IDS03, and DP03 were removed. The removal of these items was consistent with our initial theoretical assumptions about the factors. Following the model adjustment, the requirements for convergent validity were met (factor 2: AVE_before_=0.493, AVE_after_=0.530; factor 6: AVE_before_=0.467, AVE_after_=0.535; factor 7: AVE_before_=0.485, AVE_after_=0.586).

**Figure 1:**
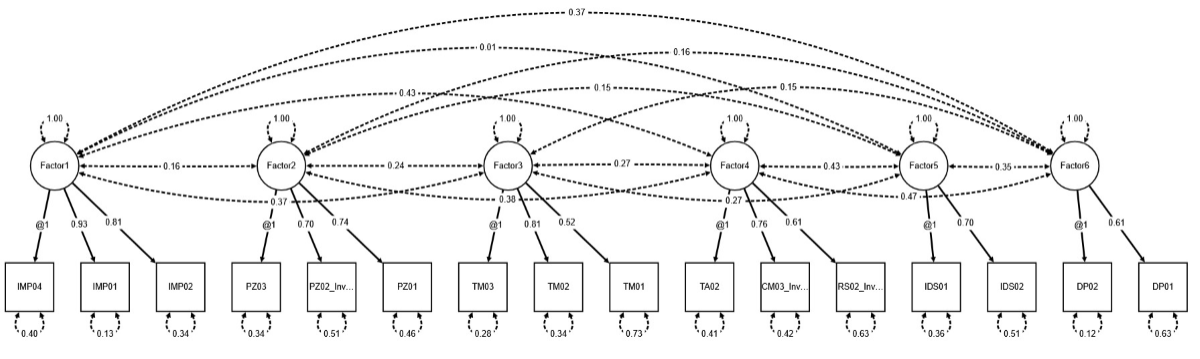
Path diagram illustrating the final CFA with standardized factor loadings, correlations, and SEs. CFA: confirmatory factor analysis.

As discriminant validity remained only partially fulfilled, we conducted a new EFA, this time based on a six-factor model. As a result, the items from factor 2 (practice culture) and factor 7 (digitally supported processes) were merged into a single factor. However, this restructuring led to deterioration in the model fit, and the newly merged factor continued to show high correlations with other factors. We therefore concluded that the items within factor 2 (practice culture) lacked sufficient conceptual distinction from the other factors. We explain this by noting that practice culture shapes all activities within a practice, provides a clear identity, and thereby guides everyday actions. If digitalization is an integral part of the enacted practice culture, it also influences factors such as process digitalization, the competencies and responsibilities of practice staff, and measures for implementing IT security and data protection. Based on the empirical findings and the substantively understandable high correlations, we therefore decided to remove factor 2 entirely. The path diagram for the final CFA is presented in Figure 1.

Following these adjustments, the requirements for discriminant validity were met. Overall, the factors showed clear distinctions from one another. The highest correlation was observed between factor 4 (staff competencies and sense of responsibility) and factor 6 (digitally supported processes), with a value of 0.469. In addition, the model fit improved substantially. An overview of the model fit for each estimated measurement model is provided in Table 6.

**Table 6:** Model fit of different estimated measurement models.

Model fit index	Model values			
	7-Factor model without adjustment	7-Factor model with adjustment	6-Factor model according to second EFA^a^	Final 6-factor model without “practice culture” factor
Robust CFI^b^	0.945	0.977	0.939	0.993
Robust TLI^c^	0.933	0.970	0.928	0.990
Robust RMSEA^d^	0.049	0.035	0.052	0.022
PCLOSE^e^	.52	.92	.36	.98
SRMR^f^	0.063	0.047	0.062	0.043

^a^EFA: exploratory factor analysis.

^b^CFI: comparative fit index.

^c^TLI: Tucker-Lewis index.

^d^RMSEA: root mean square error of approximation.

^e^PCLOSE: *P* value of close fit.

^f^SRMR: standardized root mean rquare residual.

Ultimately, the final model resulted in a questionnaire designed to measure digital maturity, consisting of 6 dimensions and 16 items (see Table 7). The final questionnaire was translated from German into English; the original questions can be found in Multimedia Appendix 4.

**Table 7:** Final questionnaire to measure the digital maturity of GP^a^ practices.

Dimension	Items
Effects of digitalization	In my medical practice, digitalization has a positive impact on the quality of patient care. In my medical practice, digitalization has a positive effect on patient satisfaction. In my medical practice, digitalization has a positive effect on the practice’s business results.
Participation of practice staff	When decisions about digitalization projects are pending, the practice staff has sufficient opportunity to contribute. In my medical practice, the practice owners make decisions about digitalization projects without involving the practice staff.^b^ Important decisions regarding digitalization projects are made jointly in our medical practice.
Maturity of the practice management system	In my medical practice, the practice management system is up to date. In my medical practice, the practice management system is easy to use. In my medical practice, the practice management system is reliable and stable.
Staff competencies and sense of responsibility	In my medical practice, the team finds it easy to learn how to use digital apps. In my medical practice, there is often a sense that no one feels responsible for digitalization projects.b In my medical practice, the staff often feels overwhelmed by new demands related to digitalization.b
IT security and data protection	In my medical practice, measures are taken to meet IT security requirements. In my medical practice, measures are taken to meet data protection requirements.
Digitally supported processes	In my medical practice, core processes (eg, appointment management, patient intake, medical history, diagnostics, treatment, documentation) are digitally supported. In my medical practice, administrative processes (eg, finance, human resources, procurement, internal communication) are digitally supported.

^a^GP: general practitioner.

^b^The data for the items will be inverted.

The questionnaire showed good internal consistency (overall Cronbach α=.809). Cronbach α values for the dimensions were .871 for effects of digitalization, .789 for participation of practice staff, .762 for maturity of the practice management system, .751 for staff competencies and sense of responsibility, .717 for IT security and data protection, and .729 for digitally supported processes.

### Results of the Maturity Level Measurement

The results of the maturity level measurements for the sample can be seen in Multimedia Appendix 5.

The overall digital maturity of the GP practices averaged 3.77 out of 5 (SD 0.50). The practices achieved their highest score in the “IT security and data protection” dimension, with a mean of 4.45 (SD 0.61). The dimensions “staff competencies and sense of responsibility” (mean 4.09, SD 0.82) and “digitally supported processes” (mean 3.99, SD 0.89) also received relatively high scores. In contrast, the lowest maturity level was observed in the “effects of digitalization” dimension (mean 3.10, SD 1.00).

## Discussion

### Principal Findings

In this study, we explored the research question of which dimensions of digital maturity in GP practices can be identified and validated to support the development of a questionnaire for measuring digital maturity. Maturity assessments for digitalization in health care are gaining increasing international traction; however, their development and level of research vary significantly across different care sectors. Our aim was to advance maturity measurements in outpatient care, both in research and in practice. The study introduces the first questionnaire for assessing digital maturity in this context, scientifically validated through psychometric testing. Through exploratory and confirmatory factor analyses, we were able to identify and confirm that the digital maturity of GP practices can be measured using 6 dimensions comprising 16 items. The questionnaire we developed captures various factors that influence the level of digitalization in a medical practice, including aspects related to practice staff, the practice management system, IT security and data protection, and digitally supported processes. In addition, the questionnaire incorporates outcome-oriented indicators, defining digital maturity in terms of the positive effects of digitalization on the medical practice. Applied to our sample, we found an average overall digital maturity score of 3.77 out of 5. Among the six dimensions, “effects of digitalization” was rated the lowest (mean 3.10, SD 1.00), while “IT security and data protection” received the highest rating (mean 4.45, SD 0.61).

### Comparison With Prior Work

A comparison of our questionnaire with existing maturity level measurements in outpatient care is currently possible only to a limited extent, as these use different measurement approaches and, in some cases, rely on dimensions originally developed for the inpatient sector. Nevertheless, both similarities and differences can be identified. The few existing studies share the understanding that digital maturity is a complex interplay of multiple dimensions. This counters the frequently voiced criticism of a narrow focus on technological aspects, which is particularly evident in maturity assessments within the hospital sector [45]. It becomes apparent that the identified dimensions can primarily be categorized into structures, processes, and outcomes, which conceptually aligns with Donabedian’s quality model [46]. The emphasis here is on structures, referring mainly to the technical infrastructure as well as the organizational and human resources and competencies of the medical practice. Although we used the dimension “staff competencies and sense of responsibility” in our study, other authors have referred to similar constructs using terms such as “resources and ability (individual),” “resources and ability (organizational),” or “capabilities, willingness, and readiness” [13,22]. An example of an outcome-oriented indicator can be found in the work of Teixeira et al [22], who examined whether digital systems have positive effects on patients, organizational structures, processes, or the financial performance of medical practices.

Unlike other maturity measurements, factors such as the interoperability of technical systems and the frequency of use are not captured in our questionnaire [13,20-22]. However, a closer analysis reveals that the frequency of use was originally part of the “practice culture” factor but was excluded during the CFA. Additionally, our questionnaire does not include aspects related to management, governance, and strategy. At most, some indications of this can be found in the factor “participation of practice staff” as an expression of staff leadership, as well as in the eliminated factor “practice culture” through questions on the practice’s mission statement. The absence of such aspects stands in contrast to maturity assessments in outpatient care [20] and, in particular, to maturity models in inpatient care, such as DigitalRadar [15,17,47]. On the one hand, this is due to the fact that variables from these subject areas were excluded during the EFA, as no underlying common factor could be identified. On the other hand, our results are based on prior expert interviews, in which these areas were considered less relevant compared to other dimensions. In this study, with regard to the limited consideration of dimensions related to management activities, we have already pointed out that in our view, this is due to the fact that the organizational form and size of a medical practice are not comparable to larger institutions, such as hospitals, where management plays a much greater role. In Germany, the solo practice remains the most common form of establishment for general practices in outpatient care, accounting for about two-thirds of all practice types [48]. Furthermore, there are also differences between maturity measurements in terms of the overall digital maturity score and the method used to calculate it. Similar to Weik et al [20], we used an overall score based on the mean values of the individual dimensions. Teixeira et al [22] used a point-based approach in which each dimension was represented by a single statement. Agreement with the statement resulted in the allocation of 1 point, and the total number of points determined the maturity level [22]. Azar et al [13] expanded their maturity measurement compared to our approach by integrating qualitative elements, specifically by defining distinct maturity levels. GP practices were assigned to a given level once a certain score threshold was reached [13].

Finally, there is also a difference in respondents completing the questionnaire. Although in our model, only GPs assessed digital maturity, the Azar et al [13] maturity model surveys practice managers and regional service officers. In a German project aimed at measuring maturity levels, medical assistants are trained as “digi-managers” through a specialized training program designed to assess the digital maturity of medical practices [11,12].

### Practical Implications

The questionnaire serves as an initial tool for GPs to assess the digitalization status of their practice. The results provide an impetus for reflecting on the current state. It can be used to systematically record the practice’s level of digitalization and to identify targeted areas for further development, such as infrastructure or the competencies of practice staff. Additionally, it can serve as a foundation for internal team discussions or for developing digitalization strategies at the practice level. By assessing their level of digitalization, multiple practices can also engage in benchmarking with one another. The results may further stimulate communication between practices, fostering improvements in their own digital maturity. For policymakers, the questionnaire offers a standardized and validated measurement instrument that quantifies digital maturity at the practice level. It can be incorporated into needs assessments, funding programs, or evaluation initiatives to track digitalization progress and to plan targeted support measures. In Germany, there are increasing calls for government funding programs to promote the digitalization of medical practices as part of a proposed Practice Future Act [49]. In this context, maturity assessments using our questionnaire could be implemented in a manner similar to DigitalRadar established under the Hospital Future Act. Finally, the questionnaire also opens up new research opportunities. It can be used in empirical studies, for example, to explore correlations between digital maturity and the successful implementation of new digital systems. Even now, there are already initial approaches that examine the connections between digital maturity and constructs such as organizational commitment and job satisfaction [50].

### Limitations

In addition to the fact that the questionnaire was validated in German, this study is subject to several limitations related to the sample, the factor-analytical model, and the methodological approach used to validate the measurement dimensions. First, the sample size of 201 participating GPs is relatively small, which limits the validity and generalizability of the results. The literature offers various recommendations regarding sample size, including guidelines on the overall sample as well as the ratio of cases to variables involved in factor analysis [51]. Independent of these recommendations, however, there remains the question of how to address potential biases resulting from an insufficient sample size. Therefore, to address this issue, we applied CFA fit indices specifically recommended for small sample sizes, following the guidance of Gaskin et al [39]. Furthermore, selection bias cannot be ruled out, as it is likely that mainly individuals open to digitalization participated in the survey. Our sample was also not representative of health care providers with respect to gender, as it included a higher proportion of male participants (61.3 %) [52]. Additionally, the influence of social desirability cannot be completely ruled out, which may have led to slightly higher ratings for factors such as “IT security and data protection” and “participation of practice staff.” Nevertheless, we believe that the use of multiple recruitment channels allowed us to reach a diverse group of participants. Regarding the sample size, the recommendations of Gaskin et al [39] were considered when interpreting the model quality, enabling an appropriate classification of the results within the given methodological constraints.

Second, there are limitations in the factor-analytical model applied. Two factors were represented by only two indicators each, which contradicts current recommendations that require at least three indicators per factor [53]. This can result in the corresponding factors being weakly identified or producing unstable parameter estimates. The content validity of such factors must also be critically evaluated. However, in the context of this study, the reduction to two indicators per factor was driven by methodological considerations. The affected items were removed due to insufficient convergent and discriminant validity. Furthermore, the theoretical foundation of the respective constructs aligned with the resulting structure, so the remaining factors were content-wise justified despite these formal limitations.

Finally, a limitation arises from the fact that the same dataset was used for both exploratory factor identification and confirmatory validation of the measurement dimensions. As a result, the independence of exploration and confirmation was constrained. This approach can lead to an overly optimistic model fit, thereby potentially compromising the validity of the results. The model could thus fit this particular data set better but perform worse on new data. The generalizability of the results is therefore limited. The decision to use the same dataset was made for practical reasons and is understandable, given the early development stage of the measurement instrument. The aim of this study was not to perform a final validation but rather to initially identify and empirically test a theoretically sound construct structure of digital maturity. Future research should focus on validating the scales using independent samples.

### Further Development of the Questionnaire

A key starting point for the further development of the questionnaire is improving the item quality and the validity of the assessed dimensions. Two dimensions, in particular, are currently based on only two items each, which limits their content coverage and statistical robustness. To better capture the full scope of the constructs and enhance measurement accuracy, these dimensions should be expanded with additional, precise, and empirically validated items.

Furthermore, new dimensions could be incorporated into the questionnaire. Specifically, aspects such as the interoperability of technical systems and topics related to governance and strategy, which have not yet been addressed, could be considered. To further improve the questionnaire’s quality, additional research should be conducted on relevant digital maturity dimensions and the findings integrated into future revisions.

When expanding the questionnaire, however, care must be taken to ensure that it remains user-friendly and that the completion time does not increase disproportionately. One potential enhancement could be to supplement the maturity level results—based on Azar et al [13]—with qualitative statements. This would provide users with a more nuanced understanding of the digital maturity level, rather than a purely numerical evaluation. In addition, consideration should be given to not restricting the questionnaire completion exclusively to GPs. As demonstrated in previous studies, including other health care providers can be valuable to obtain a more comprehensive perspective and enhance the quality of the collected data [12,13]. Finally, it appears prudent to extend the questionnaire to other specialist areas within outpatient care. Our study has so far focused solely on GP practices, while specialist practices have not been included. Therefore, the adaptation and validation of the questionnaire for this target group should be explored.

### Conclusion

This study is the first empirical investigation to identify and validate the dimensions of digital maturity in GP practices. The central outcome is a scientifically robust questionnaire that provides a foundation for future maturity assessments. The findings demonstrate that digital maturity is a multidimensional construct that can be captured through a variety of technical, human, and organizational factors. Outcome-oriented factors, such as enhanced patient satisfaction, can also serve as indicators of a high level of digital maturity in medical practices. This study offers practical implications for GPs, policymakers, and the scientific community. In future research, the developed questionnaire should be further refined, validated, and adapted to different contexts. Overall, the current results contribute to the establishment of maturity assessments in the outpatient sector and can serve as a basis for supporting the digital transformation in a targeted and sustainable way through accompanying evaluations.

## Appendix

Multimedia Appendix 1 Completed Checklist for Reporting Results of Internet E-Surveys (CHERRIES).

Multimedia Appendix 2 Survey questionnaire for general practitioners.

Multimedia Appendix 3 Items and abbreviations used.

Multimedia Appendix 4 Final questionnaire (original version).

Multimedia Appendix 5 Digital maturity by dimension and as an overall value (mean, SD).

## Abbreviations

AVE: average variance extracted
CFA: confirmatory factor analysis
CFI: comparative fit index
CHERRIES: Checklist for Reporting Results of Internet E-Surveys
EFA: exploratory factor analysis
GP: general practitioner
KMO: Kaiser-Meyer-Olkin
PCLOSE: *P* value of close fit
RMSEA: root mean square error of approximation
SRMR: standardized root mean square residual
TLI: Tucker-Lewis index

## Data Availability

The data supporting this study’s findings are available from the corresponding author upon reasonable request.
