# Supplementary material for: Development and Validation of a Questionnaire to Measure Digital Maturity of General Practitioner Practices: Web-Based Cross-Sectional Survey Study
Source: J Med Internet Res. 2025 Oct 14;27:e81416. doi: 10.2196/81416 (PMC12569491; doi:10.2196/81416)
Supplement: Multimedia Appendix 2 [file jmir_v27i1e81416_app2.docx]

**Multimedia Appendix 2: Survey questionnaire for general practitioners.**

**English Version**

**Introduction:**

Dear Sir or Madam,

Thank you for your interest in this survey.

**The content of the survey:**

The healthcare system is becoming increasingly digital and is set to become more digital. On the one hand, digitalization is associated with opportunities, for example for the quality of patient care. On the other hand, it is often associated with additional work. We are interested in your perspective as GPs.

As part of a dissertation at the Chair of Health Informatics at the Faculty of Health at Witten/Herdecke University, we are investigating which factors can be used to assess the level of digitalization in German GP practices. In this context, we are also interested in your motivation to survey your own level of digitalization.

We understand digitalization as the digital transformation that takes into account organizational and social aspects in addition to the introduction of IT technologies. For us, digitalization in medical practices does not just mean converting analogue processes into digital ones. It is associated with conscious change processes.

**The research questions of this survey are:**

**1. what are the motives of GPs to survey the digitalization status of their practice?**

**2. which factors should be used to assess the level of digitalization of GP practices?**

**3. which factors actually influence the level of digitalization of GP practices?**

**Organizational information about the survey:**

This survey is aimed at general practitioners in Germany. It will take about 20 minutes to complete the survey. We would like to ask you to answer the questionnaire completely.

This research project has been approved by the Ethics Committee of Witten/Herdecke University (S-47/2023). Under "Show privacy policy" you will find all the necessary information on data processing and your rights as a participant. In order to participate in this survey, it is necessary that you accept the privacy policy.

*Show privacy policy*

**Section 1: Demographic data and information on the medical practice**

To begin with, we would like to find out more about you as a person and about your medical practice.

1. **Which gender do you feel you belong to?**

Female

Male

Nonbinary

no gender

no answer

other

### **2. How old are you?**

Please enter an integer value

### **3. What type of practice do you work in?**

Single practice

Joint professional practice

Group practice

Medical care center

### **4. Where do you work as a general practitioner? I work in a place with...**

less than 5,000 inhabitants

5,000 to 20,000 inhabitants

20,001 to 100,000 inhabitants

100,001 to 500,000 inhabitants

more than 500,000 inhabitants

### **5. On a scale of 0 to 5, how high would you rate the average digital health literacy of your patients?**

Note: By digital health literacy, we mean the ability to find, understand, assess and use health-related information in relation to digital applications and digital information services. 0 = Patients are not at all able to use digital applications and digital information services in a self-determined and health-oriented manner. 5 = Patients are fully able to use digital applications and digital information services in a self-determined and health-oriented manner. Please use the slider.

### **6. On a scale from 0 to 10, how high would you rate the current level of digitalization in your practice?**

Please use the slider.

## **Section 2: Information on dimensions of digital maturity (i.e. people, organization, technology)**

### **Digitally supported processes**

In the following, we would like to know whether and to what extent processes in your practice are already digitally supported.

Please indicate how much you agree with the following statements. You can rate your answers from " Strongly disagree " to "Strongly agree".

|  | Strongly disagree | Disagree | Neutral | Agree | Strongly agree |
| --- | --- | --- | --- | --- | --- |
| In my medical practice, the core processes (e.g. appointment management, patient reception, anamnesis, diagnostics, treatment, documentation) are digitally supported. | 🞎 | 🞎 | 🞎 | 🞎 | 🞎 |
| In my medical practice, administrative processes (e.g. finance, personnel, purchasing, internal communication) are supported digitally. | 🞎 | 🞎 | 🞎 | 🞎 | 🞎 |
| In my medical practice, the exchange with external service providers and institutions (e.g. specialists, laboratories, health insurance companies) is digitally supported. | 🞎 | 🞎 | 🞎 | 🞎 | 🞎 |

### **Skills and abilities of the practice team**

In the following, we would like to find out more about the practice team in your medical practice.

Please indicate how much you agree with the following statements. You can rate your answers from "Strongly disagree" to "Strongly agree".

Note: We use the term digital applications in the questions. By this we mean, among other things, the use of the practice management system, applications of the telematics infrastructure, digital care services and telemedical services, as well as the use of networked medical devices and artificial intelligence.

|  | Strongly disagree | Tends not to apply | Part/Part | Tends to apply | Strongly agree |
| --- | --- | --- | --- | --- | --- |
| In my medical practice, the practice team is familiar with digital applications. | 🞎 | 🞎 | 🞎 | 🞎 | 🞎 |
| In my medical practice, it is easy for the practice team to learn how to use digital applications. | 🞎 | 🞎 | 🞎 | 🞎 | 🞎 |
| In my medical practice, the practice team enjoys using digital applications. | 🞎 | 🞎 | 🞎 | 🞎 | 🞎 |
| I regularly find out about new digital applications for medical practices. | 🞎 | 🞎 | 🞎 | 🞎 | 🞎 |
| In my medical practice, the practice team sees the use of digital applications as an important part of their profession. | 🞎 | 🞎 | 🞎 | 🞎 | 🞎 |
| In my medical practice, the use of digital applications is a matter of course for the practice team. | 🞎 | 🞎 | 🞎 | 🞎 | 🞎 |

### **Organizational structures in the medical practice**

In the following, we would like to learn more about the organizational structures in your medical practice.

Please indicate how much you agree with the following statements. You can rate your answers from "Strongly disagree" to "Strongly agree".

Note: We use the term digital applications in the questions. By this we mean, among other things, the use of the practice management system, applications of the telematics infrastructure, digital care services and telemedical services, as well as the use of networked medical devices and artificial intelligence.

|  | Strongly disagree | Disagree | Neutral | Agree | Strongly agree |
| --- | --- | --- | --- | --- | --- |
| In my medical practice, we have clear responsibilities for digitalization projects in the practice team. | 🞎 | 🞎 | 🞎 | 🞎 | 🞎 |
| In my medical practice, I often get the feeling that nobody feels responsible for digitalization projects. | 🞎 | 🞎 | 🞎 | 🞎 | 🞎 |
| In my medical practice, digitalization projects are generally well coordinated. | 🞎 | 🞎 | 🞎 | 🞎 | 🞎 |
| In my medical practice, the use of digital applications is part of our mission statement. | 🞎 | 🞎 | 🞎 | 🞎 | 🞎 |
| In my medical practice, digital applications are always used if the situation makes it possible. | 🞎 | 🞎 | 🞎 | 🞎 | 🞎 |
| In my medical practice, the use of digital applications is part of everyday practice life. | 🞎 | 🞎 | 🞎 | 🞎 | 🞎 |
| When decisions on digitalization projects are pending, practice staff have ample opportunity to get involved. | 🞎 | 🞎 | 🞎 | 🞎 | 🞎 |
| In my medical practice, practice owners make decisions on digitalization projects over the heads of the practice staff. | 🞎 | 🞎 | 🞎 | 🞎 | 🞎 |
| Important decisions on digitalization projects are made jointly in our medical practice. | 🞎 | 🞎 | 🞎 | 🞎 | 🞎 |
| In my medical practice, there is a high willingness to change when it comes to new requirements in connection with digitalization. | 🞎 | 🞎 | 🞎 | 🞎 | 🞎 |
| In my medical practice, patients are interviewed to identify external requirements in connection with digitalization. | 🞎 | 🞎 | 🞎 | 🞎 | 🞎 |
| In my medical practice, the practice staff react to new requirements in connection with digitalization with a lot of excessive demands. | 🞎 | 🞎 | 🞎 | 🞎 | 🞎 |
| In my medical practice, current problems with the use of digital applications, their causes and possible suggestions for improvement are discussed within the practice team. | 🞎 | 🞎 | 🞎 | 🞎 | 🞎 |
| In my medical practice, processes for the use of digital applications are documented in writing and are available digitally for inspection by the practice staff. | 🞎 | 🞎 | 🞎 | 🞎 | 🞎 |
| The use of digital applications is standardized in my medical practice. | 🞎 | 🞎 | 🞎 | 🞎 | 🞎 |

### **Technical maturity of the medical practice**

In the following, we would like to learn more about the technical equipment (e.g. the practice management system and medical devices) in your practice.

Please indicate how much you agree with the following statements. You can rate your answers from "Strongly disagree" to "Strongly agree".

|  | Strongly disagree | Disagree | Neutral | Agree | Strongly agree |
| --- | --- | --- | --- | --- | --- |
| The practice management system in my medical practice is up to date. | 🞎 | 🞎 | 🞎 | 🞎 | 🞎 |
| The practice management system is easy to use in my medical practice. | 🞎 | 🞎 | 🞎 | 🞎 | 🞎 |
| The practice management system in my medical practice is reliable and stable. | 🞎 | 🞎 | 🞎 | 🞎 | 🞎 |
| In my medical practice, medical devices (e.g. ECG, ultrasound, scales) are connected to the practice management system via digital interfaces. | 🞎 | 🞎 | 🞎 | 🞎 | 🞎 |
| My practice management system only allows data exchange with manufacturer-specific medical devices (e.g. ECG, ultrasound, scales). | 🞎 | 🞎 | 🞎 | 🞎 | 🞎 |
| In my medical practice, employees receive training on the secure handling of patient data when using IT systems. | 🞎 | 🞎 | 🞎 | 🞎 | 🞎 |
| In my medical practice, measures are taken to meet data protection compliance requirements. | 🞎 | 🞎 | 🞎 | 🞎 | 🞎 |
| In my medical practice, measures are taken to meet IT security compliance requirements. | 🞎 | 🞎 | 🞎 | 🞎 | 🞎 |
| My medical practice has a high-performance Internet connection (e.g. in terms of data transfer rate). | 🞎 | 🞎 | 🞎 | 🞎 | 🞎 |

### **Effects of digitalization in the medical practice**

|  | Strongly disagree | Disagree | Neutral | Agree | Strongly agree |
| --- | --- | --- | --- | --- | --- |
| In my medical practice, digitalization has a positive impact on the quality of patient care. | 🞎 | 🞎 | 🞎 | 🞎 | 🞎 |
| In my medical practice, digitalization has a positive effect on patient satisfaction. | 🞎 | 🞎 | 🞎 | 🞎 | 🞎 |
| In my medical practice, digitalization has a positive effect on the workload of the practice staff. | 🞎 | 🞎 | 🞎 | 🞎 | 🞎 |
| In my medical practice, digitalization has a positive effect on the practice's business results. | 🞎 | 🞎 | 🞎 | 🞎 | 🞎 |

## **Further comments**

Finally, we would like to give you the opportunity to send us further thoughts on digitalization in outpatient care, especially for GP practices.

What other aspects of the digitalization status of GP practices in outpatient care are of general interest to you or would you like to share with us?

Please use the free text field.

## **Conclusion and goodbye**

Thank you very much for your participation!

You have reached the end of this survey. Your answers have now been recorded.

Do you have any colleagues who might also be interested in taking part? If so, we would be delighted if you could forward the *survey link*

You can now close the browser window.

**Deutsche Version**

**Einleitung**

Sehr geehrte Damen und Herren,

vielen Dank für Ihr Interesse an dieser Umfrage.

**Zum Inhalt der Umfrage:**

Das Gesundheitswesen wird immer digitaler und soll digitaler werden. Auf der einen Seite wird die Digitalisierung mit Chancen, beispielsweise für die Qualität der Patientenversorgung, verbunden. Auf der anderen Seite wird sie häufig mit Mehraufwand assoziiert. **Uns interessiert Ihre Perspektive als Hausärztinnen und Hausärzte.**

Im Rahmen einer Dissertation am Lehrstuhl für Gesundheitsinformatik an der Fakultät für Gesundheit der Universität Witten/Herdecke untersuchen wir, anhand welcher Faktoren der Digitalisierungsstand deutscher Hausarztpraxen erhoben werden kann. In diesem Zusammenhang interessiert uns auch Ihre Motivation zur Erhebung des eigenen Digitalisierungsstands.

Wir verstehen unter Digitalisierung die digitale Transformation, die neben der Einführung von IT-Technologien auch organisatorische und gesellschaftliche Aspekte berücksichtigt. Digitalisierung in Arztpraxen bedeutet für uns also nicht nur die Umwandlung von analogen in digitale Prozesse. Sie ist mit bewussten Veränderungsprozessen verbunden.

**Die Forschungsfragen dieser Umfrage lauten:**

**1. Was sind die Motive von Hausärztinnen und Hausärzte zur Erhebung des Digitalisierungsstands ihrer
Praxis?**

**2. Anhand welcher Faktoren sollte der Digitalisierungsstand von Hausarztpraxen erhoben werden?**

**3. Welche Faktoren beeinflussen tatsächlich den Digitalisierungsstand von Hausarztpraxen?**

**Organisatorisches zur Umfrage:**

Diese Umfrage richtet sich an praktizierende Hausärztinnen und Hausärzte in Deutschland. Das Ausfüllen der Umfrage wird etwa 20 Minuten in Anspruch nehmen. Wir möchten Sie bitten, den Fragebogen vollständig zu beantworten.

Dieses Forschungsprojekt ist von der Ethikkommission der Universität Witten/Herdecke genehmigt worden (S-47/2023). Unter "Datenschutzerklärung anzeigen" finden Sie alle notwendigen Informationen zur Datenverarbeitung und zu Ihren Rechten als Teilnehmerin und Teilnehmer. Um an dieser Umfrage teilnehmen zu können, ist es notwendig, dass Sie die Datenschutzerklärung akzeptieren.

*Datenschutzerklärung anzeigen*

## **Abschnitt 1: Demografische Daten und Angaben zur Praxis**

Zu Beginn möchten wir mehr über Sie als Person und zu Ihrer Arztpraxis erfahren.

1. **Welchem Geschlecht fühlen Sie sich zugehörig?**

Weiblich

Männlich

Nicht-Binär

Kein Geschlecht

Keine Angabe

Sonstiges

### **Wie alt sind Sie?**

Bitte verwenden Sie einen ganzzahligen Wert (z. B. 40)

### **In welcher Praxisform sind Sie tätig?**

Einzelpraxis

Praxisgemeinschaft

Berufsausübungsgemeinschaft (BAG)

Medizinisches Versorgungszentrum (MVZ)

### **Wo arbeiten Sie als Hausärztin/Hausarzt? Ich arbeite in einem Ort mit...**

weniger als 5.000 Einwohnern

5.000 bis 20.000 Einwohnern

20.001 bis 100.000 Einwohnern

100.001 bis 500.000 Einwohnern

mehr als 500.000 Einwohnern

### **Wie hoch schätzen Sie die durchschnittliche digitale Gesundheitskompetenz Ihrer Patientinnen und Patienten auf einer Skala von 0 bis 5 ein?**

**Hinweis**: Unter digitaler Gesundheitskompetenz verstehen wir die Fähigkeit, gesundheitsrelevante Informationen in Bezug auf digitale Anwendungen und digitale Informationsangebote finden, verstehen, beurteilen und anwenden zu können.

0 = Patientinnen und Patienten können digitale Anwendungen und digitale Informationsangebote überhaupt nicht selbstbestimmt und gesundheitsorientiert einsetzen.

5 = Patientinnen und Patienten können digitale Anwendungen und digitale Informationsangebote voll und ganz selbstbestimmt und gesundheitsorientiert einsetzen.

**Nutzen Sie bitte den Schieberegler.**

### **Wie hoch schätzen Sie den aktuellen Digitalisierungsstand Ihrer Praxis auf einer Skala von 0 bis 10 ein?**

**Nutzen Sie hierzu bitte den Schieberegler.**

## **Abschnitt 2: Informationen über die Dimensionen des digitalen Reifegrads (d. h. Menschen, Organisation, Technologie)**

### **Digital unterstützte Prozesse**

Im Folgenden möchten wir wissen, ob und in welchem Umfang Prozesse in Ihrer Praxis bereits digital unterstützt werden.

Bitte geben Sie an, wie sehr Sie den folgenden Aussagen zustimmen. Sie können Ihre Antworten von „trifft überhaupt nicht zu“ bis „trifft voll und ganz zu“ bewerten.

|  | Trifft überhaupt nicht zu | Trifft eher nicht zu | Teils/Teils | Trifft eher zu | Trifft voll und ganz zu |
| --- | --- | --- | --- | --- | --- |
| In meiner Arztpraxis sind die Kernprozesse (z. B. Terminmanagement, Patientenannahme, Anamnese, Diagnostik, Behandlung, Dokumentation) digital unterstützt. | 🞎 | 🞎 | 🞎 | 🞎 | 🞎 |
| In meiner Arztpraxis sind administrative Prozesse (z. B. Finanzen, Personal, Einkauf, interne Kommunikation) digital unterstützt. | 🞎 | 🞎 | 🞎 | 🞎 | 🞎 |
| In meiner Arztpraxis ist der Austausch mit externen Leistungserbringenden und Institutionen (z. B. Fachärztinnen und Fachärzten, Laboren, Krankenkassen) digital unterstützt. | 🞎 | 🞎 | 🞎 | 🞎 | 🞎 |

### **Fähig- und Fertigkeiten vom Praxisteam**

Im Folgenden möchten wir mehr über das Praxisteam in Ihrer Arztpraxis erfahren.

Bitte geben Sie an, wie sehr Sie den folgenden Aussagen zustimmen. Sie können Ihre Antworten von „Trifft überhaupt nicht zu“ bis „Trifft voll und ganz zu“ bewerten.

Hinweis: Wir verwenden in den Fragen den Begriff digitale Anwendungen. Darunter verstehen wir u.a. die Nutzung des Praxisverwaltungssystems, Anwendungen der Telematikinfrastruktur, digitale Pflegedienste und telemedizinische Dienste sowie den Einsatz von vernetzten medizinischen Geräten und künstlicher Intelligenz.

|  | Trifft überhaupt nicht zu | Trifft eher nicht zu | Teils/Teils | Trifft eher zu | Trifft voll und ganz zu |
| --- | --- | --- | --- | --- | --- |
| In meiner Arztpraxis kennt sich das Praxisteam mit digitalen Anwendungen aus. | 🞎 | 🞎 | 🞎 | 🞎 | 🞎 |
| In meiner Arztpraxis fällt es dem Praxisteam leicht, die Bedienung von digitalen Anwendungen zu lernen. | 🞎 | 🞎 | 🞎 | 🞎 | 🞎 |
| In meiner Arztpraxis macht es dem Praxisteam Spaß, digitale Anwendungen zu nutzen. | 🞎 | 🞎 | 🞎 | 🞎 | 🞎 |
| Ich informiere mich regelmäßig über neue digitale Anwendungen für Arztpraxen. | 🞎 | 🞎 | 🞎 | 🞎 | 🞎 |
| In meiner Arztpraxis ist das Praxisteam davon überzeugt, dass die Nutzung von digitalen Anwendungen den persönlichen Kontakt zwischen den Menschen verringert. | 🞎 | 🞎 | 🞎 | 🞎 | 🞎 |
| In meiner Arztpraxis ist das Praxisteam davon überzeugt, dass die Nutzung von digitalen Anwendungen vieles umständlicher macht. | 🞎 | 🞎 | 🞎 | 🞎 | 🞎 |

### **Organisatorische Strukturen in der Arztpraxis**

Im Folgenden möchten wir mehr über die Organisationsstrukturen in Ihrer Arztpraxis erfahren und bitten Sie, uns mitzuteilen, wie sehr Sie den folgenden Aussagen zustimmen.

Sie können Ihre Antworten von „Trifft überhaupt nicht zu“ bis „Trifft voll und ganz zu“ bewerten.

Hinweis: Wir verwenden in den Fragen den Begriff digitale Anwendungen. Darunter verstehen wir u.a. den Einsatz des Praxisverwaltungssystems, Anwendungen der Telematikinfrastruktur, digitale Pflegedienste und telemedizinische Dienste sowie den Einsatz von vernetzten medizinischen Geräten und künstlicher Intelligenz.

|  | Trifft überhaupt nicht zu | Trifft eher nicht zu | Teils/Teils | Trifft eher zu | Trifft voll und ganz zu |
| --- | --- | --- | --- | --- | --- |
| In meiner Arztpraxis haben wir im Praxisteam klare Zuständigkeiten für Digitalisierungsvorhaben. | 🞎 | 🞎 | 🞎 | 🞎 | 🞎 |
| In meiner Arztpraxis hat man oft das Gefühl, dass sich niemand für Digitalisierungsvorhaben verantwortlich fühlt. | 🞎 | 🞎 | 🞎 | 🞎 | 🞎 |
| In meiner Arztpraxis sind Digitalisierungsvorhaben in der Regel gut koordiniert. | 🞎 | 🞎 | 🞎 | 🞎 | 🞎 |
| In meiner Arztpraxis ist die Nutzung digitaler Anwendungen Teil unseres Praxisleitbildes. | 🞎 | 🞎 | 🞎 | 🞎 | 🞎 |
| In meiner Arztpraxis werden stets digitale Anwendungen genutzt, sofern die Situation den Einsatz möglich macht. | 🞎 | 🞎 | 🞎 | 🞎 | 🞎 |
| In meiner Arztpraxis wird die Nutzung von digitalen Anwendungen im Praxisalltag gelebt. | 🞎 | 🞎 | 🞎 | 🞎 | 🞎 |
| Wenn Entscheidungen zu Digitalisierungsvorhaben anstehen, hat das Praxispersonal ausreichend Gelegenheit, sich einzubringen. | 🞎 | 🞎 | 🞎 | 🞎 | 🞎 |
| In meiner Arztpraxis treffen Praxisinhaberinnen und Praxisinhaber Entscheidungen zu Digitalisierungsvorhaben über die Köpfe des Praxispersonals hinweg. | 🞎 | 🞎 | 🞎 | 🞎 | 🞎 |
| Wichtige Entscheidungen zu Digitalisierungsvorhaben werden in unserer Arztpraxis gemeinsam getroffen. | 🞎 | 🞎 | 🞎 | 🞎 | 🞎 |
| In meiner Arztpraxis werden aktuelle Probleme bei der Nutzung digitaler Anwendungen, ihre Ursachen und mögliche Verbesserungsvorschläge im Praxisteam diskutiert. | 🞎 | 🞎 | 🞎 | 🞎 | 🞎 |
| In meiner Arztpraxis werden Prozesse zur Nutzung von digitalen Anwendungen schriftlich dokumentiert und stehen dem Praxispersonal digital zur Einsicht bereit. | 🞎 | 🞎 | 🞎 | 🞎 | 🞎 |
| In meiner Arztpraxis ist die Nutzung von digitalen Anwendungen standardisiert. | 🞎 | 🞎 | 🞎 | 🞎 | 🞎 |
| In meiner Arztpraxis besteht eine hohe Veränderungsbereitschaft bei neuen Anforderungen im Zusammenhang mit der Digitalisierung. | 🞎 | 🞎 | 🞎 | 🞎 | 🞎 |
| In meiner Arztpraxis werden Patientinnen und Patienten befragt, um externe Anforderungen im Zusammenhang mit der Digitalisierung zu identifizieren. | 🞎 | 🞎 | 🞎 | 🞎 | 🞎 |
| In meiner Arztpraxis reagiert das Praxispersonal auf neue Anforderungen im Zusammenhang mit der Digitalisierung mit viel Überforderung. | 🞎 | 🞎 | 🞎 | 🞎 | 🞎 |

### **Technische Ausstattung der Arztpraxis**

Im Folgenden möchten wir mehr über die technische Ausstattung (z.B. das Praxisverwaltungssystem und medizinische Geräte) Ihrer Praxis erfahren.

Bitte geben Sie an, wie sehr Sie den folgenden Aussagen zustimmen. Sie können Ihre Antworten von „Trifft überhaupt nicht zu“ bis „Trifft voll und ganz zu“ bewerten.

|  | Trifft überhaupt nicht zu | Trifft eher nicht zu | Teils/Teils | Trifft eher zu | Trifft voll und ganz zu |
| --- | --- | --- | --- | --- | --- |
| In meiner Arztpraxis ist das Praxisverwaltungssystem auf dem neuesten Stand. | 🞎 | 🞎 | 🞎 | 🞎 | 🞎 |
| In meiner Arztpraxis ist das Praxisverwaltungssystem einfach zu benutzen. | 🞎 | 🞎 | 🞎 | 🞎 | 🞎 |
| In meiner Arztpraxis ist das Praxisverwaltungssystem zuverlässig und stabil. | 🞎 | 🞎 | 🞎 | 🞎 | 🞎 |
| In meiner Arztpraxis sind medizinische Geräte (z. B. EKG, Ultraschall, Waage) über digitale Schnittstellen mit dem Praxisverwaltungssystem verbunden. | 🞎 | 🞎 | 🞎 | 🞎 | 🞎 |
| Mein Praxisverwaltungssystem ermöglicht den Datenaustausch nur mit herstellerspezifischen medizinischen Geräten (z. B. EKG, Ultraschall, Waage). | 🞎 | 🞎 | 🞎 | 🞎 | 🞎 |
| In meiner Arztpraxis werden Maßnahmen ergriffen, um Anforderungen zur Einhaltung der IT-Sicherheit zu erfüllen. | 🞎 | 🞎 | 🞎 | 🞎 | 🞎 |
| In meiner Arztpraxis werden Maßnahmen ergriffen, um Anforderungen zur Einhaltung des Datenschutzes zu erfüllen. | 🞎 | 🞎 | 🞎 | 🞎 | 🞎 |
| In meiner Arztpraxis erhalten Mitarbeiterinnen und Mitarbeiter für die Nutzung von IT-Systemen Schulungen zum sicheren Umgang mit Patientendaten. | 🞎 | 🞎 | 🞎 | 🞎 | 🞎 |
| Meine Arztpraxis verfügt über einen leistungsstarken Internetanschluss (z. B. im Hinblick auf die Datenübertragungsrate). | 🞎 | 🞎 | 🞎 | 🞎 | 🞎 |

### **Auswirkungen der Digitalisierung in der Arztpraxis**

Im Folgenden würden wir gerne mehr über die Auswirkungen der Digitalisierung in Ihrer Praxis erfahren.

Bitte geben Sie an, wie sehr Sie den folgenden Aussagen zustimmen. Sie können Ihre Antworten von "Trifft überhaupt nicht zu" bis "Trifft voll und ganz zu" einstufen.

|  | Trifft überhaupt nicht zu | Trifft eher nicht zu | Teils/Teils | Trifft eher zu | Trifft voll und ganz zu |
| --- | --- | --- | --- | --- | --- |
| In meiner Arztpraxis wirkt sich die Digitalisierung positiv auf die Qualität der Patientenversorgung aus. | 🞎 | 🞎 | 🞎 | 🞎 | 🞎 |
| In meiner Arztpraxis wirkt sich die Digitalisierung positiv auf die Patientenzufriedenheit aus. | 🞎 | 🞎 | 🞎 | 🞎 | 🞎 |
| In meiner Arztpraxis wirkt sich die Digitalisierung positiv auf die Arbeitsbelastung des Praxispersonals aus. | 🞎 | 🞎 | 🞎 | 🞎 | 🞎 |
| In meiner Arztpraxis wirkt sich die Digitalisierung positiv auf die betriebswirtschaftlichen Ergebnisse der Praxis aus. | 🞎 | 🞎 | 🞎 | 🞎 | 🞎 |

## **Weitere Anmerkungen**

Abschließend möchten wir Ihnen die Möglichkeit geben, uns weitere Gedanken zur Digitalisierung in der ambulanten Versorgung, speziell für Hausarztpraxen, zukommen zu lassen.

Welche weiteren Aspekte des Digitalisierungsstandes von Hausarztpraxen in der ambulanten Versorgung sind für Sie von allgemeinem Interesse oder möchten Sie uns mitteilen?

Bitte nutzen Sie das Freitextfeld.

## **Verabschiedung**

Vielen Dank für Ihre Teilnahme!

Sie haben das Ende dieser Umfrage erreicht. Ihre Antworten wurden nun erfasst.

Haben Sie Kolleginnen und Kollegen, die ebenfalls an einer Teilnahme interessiert sein könnten? Dann würden wir uns freuen, wenn Sie den Umfragelink weiterleiten. Sie können das Browserfenster nun schließen.
