# Supplementary material for: Development and Validation of a Questionnaire to Measure Digital Maturity of General Practitioner Practices: Web-Based Cross-Sectional Survey Study
Source: J Med Internet Res. 2025 Oct 14;27:e81416. doi: 10.2196/81416 (PMC12569491; doi:10.2196/81416)
Supplement: Multimedia Appendix 3 [file jmir_v27i1e81416_app3.docx]

**Multimedia Appendix 3: Items and abbreviations used**

| Item Abbreviation | Item text |
| --- | --- |
| DP01 | In my medical practice, core processes (e.g., appointment management, patient intake, medical history, diagnostics, treatment, documentation) are digitally supported. |
| DP02 | In my medical practice, administrative processes (e.g., finance, human resources, procurement, internal communication) are digitally supported. |
| DP03 | In my medical practice, communication with external service providers and institutions (e.g., specialists, laboratories, health insurance providers) is digitally supported. |
| TA01 | In my medical practice, the team is familiar with digital applications. |
| TA02 | In my medical practice, the team finds it easy to learn how to use digital applications |
| TA03 | In my medical practice, the team enjoys using digital applications. |
| TA04 | I regularly stay informed about new digital applications for medical practices. |
| RU01 | In my medical practice, the team considers the use of digital applications an important part of their profession. |
| RU02 | In my medical practice, using digital applications is naturally considered part of the team's responsibilities. |
| RS01 | In my medical practice, we have clear responsibilities within the team for digitalization projects. |
| RS02 | In my medical practice, there is often a sense that no one feels responsible for digitalization projects.^a^ |
| RS03 | In my medical practice, digitalization projects are generally well coordinated. |
| PC01 | In my medical practice, the use of digital applications is part of our mission statement. |
| PC02 | In my medical practice, digital applications are consistently used whenever the situation allows for it. |
| PC03 | In my medical practice, the use of digital applications is an integral part of daily routines. |
| PP01 | When decisions about digitalization projects are pending, the practice staff has sufficient opportunity to contribute. |
| PP02 | In my medical practice, the practice owners make decisions about digitalization projects without involving the practice staff.^a^ |
| PP03 | Important decisions regarding digitalization projects are made jointly in our medical practice. |
| CM01 | In my medical practice, there is a high willingness to adapt to new requirements related to digitalization. |
| CM02 | In my medical practice, patients are surveyed to identify external requirements related to digitalization. |
| CM03 | In my medical practice, the staff often feels overwhelmed by new demands related to digitalization.^a^ |
| KQM01 | In my medical practice, current problems related to the use of digital applications, their causes, and possible improvements are discussed within the team. |
| KQM02 | In my medical practice, processes for using digital applications are documented in writing and made digitally accessible to the staff. |
| KQM03 | In my medical practice, the use of digital applications is standardized. |
| TM01 | In my medical practice, the practice management system is up to date. |
| TM02 | In my medical practice, the practice management system is easy to use. |
| TM03 | In my medical practice, the practice management system is reliable and stable. |
| IO01 | In my medical practice, medical devices (e.g., ECG, ultrasound, scales) are connected to the practice management system via digital interfaces. |
| IO02 | My practice management system only allows data exchange with manufacturer-specific medical devices (e.g., ECG, ultrasound, scales).^a^ |
| IDS01 | In my medical practice, measures are taken to meet IT security compliance requirements. |
| IDS02 | In my medical practice, measures are taken to meet data protection compliance requirements. |
| IDS03 | In my medical practice, employees receive training on the secure handling of patient data when using IT systems. |
| NI01 | My medical practice has a high-performance internet connection (e.g., in terms of data transfer rate). |
| EFF01 | In my medical practice, digitalization has a positive impact on the quality of patient care. |
| EFF02 | In my medical practice, digitalization has a positive effect on patient satisfaction. |
| EFF03 | In my medical practice, digitalization has a positive effect on the workload of the practice staff. |
| EFF04 | In my medical practice, digitalization has a positive effect on the practice's business results. |

^a^ The data for items "RS02", "PP02", "CM03" and "IO02" were inverted.
