# Supplementary material for: Development and Validation of a Questionnaire to Measure Digital Maturity of General Practitioner Practices: Web-Based Cross-Sectional Survey Study
Source: J Med Internet Res. 2025 Oct 14;27:e81416. doi: 10.2196/81416 (PMC12569491; doi:10.2196/81416)
Supplement: Multimedia Appendix 4 [file jmir_v27i1e81416_app4.docx]

**Appendix 4:** Final questionnaire to measure digital maturity of GP practices (Original version)

| Dimension | Items^a^ |
| --- | --- |
| Auswirkungen der Digitalisierung | In meiner Arztpraxis wirkt sich die Digitalisierung positiv auf die Qualität der Patientenversorgung aus. |
|  | In meiner Arztpraxis wirkt sich die Digitalisierung positiv auf die Patientenzufriedenheit aus. |
|  | In meiner Arztpraxis wirkt sich die Digitalisierung positiv auf die betriebswirtschaftlichen Ergebnisse der Praxis aus. |
| Partizipation des Praxispersonals | Wenn Entscheidungen zu Digitalisierungsvorhaben anstehen, hat das Praxispersonal ausreichend Gelegenheit, sich einzubringen. |
|  | In meiner Arztpraxis treffen Praxisinhaberinnen und Praxisinhaber Entscheidungen zu Digitalisierungsvorhaben über die Köpfe des Praxispersonal hinweg.^b^ |
|  | Wichtige Entscheidungen zu Digitalisierungsvorhaben werden in unserer Arztpraxis gemeinsam getroffen. |
| Reife des Praxisverwaltungssystems | In meiner Arztpraxis ist das Praxisverwaltungssystem auf dem neuesten Stand. |
|  | In meiner Arztpraxis ist das Praxisverwaltungssystem einfach zu benutzen. |
|  | In meiner Arztpraxis ist das Praxisverwaltungssystem zuverlässig und stabil. |
| Kompetenzen und Verantwortungsbewusstsein des Praxispersonals | In meiner Arztpraxis fällt es dem Praxisteam leicht, die Bedienung von digitalen Anwendungen zu lernen. |
|  | In meiner Arztpraxis hat man oft das Gefühl, dass sich niemand für Digitalisierungsvorhaben verantwortlich fühlt.^b^ |
|  | In meiner Arztpraxis reagiert das Praxispersonal auf neue Anforderungen im Zusammenhang mit der Digitalisierung mit viel Überforderung.^b^ |
| IT-Sicherheit und Datenschutz | In meiner Arztpraxis werden Maßnahmen ergriffen, um Anforderungen zur Einhaltung der IT-Sicherheit zu erfüllen. |
|  | In meiner Arztpraxis werden Maßnahmen ergriffen, um Anforderungen zur Einhaltung des Datenschutzes zu erfüllen. |
| Digital unterstützte Prozesse | In meiner Arztpraxis sind die Kernprozesse (z. B. Terminmanagement, Patientenannahme, Anamnese, Diagnostik, Behandlung, Dokumentation) digital unterstützt. |
|  | In meiner Arztpraxis sind administrative Prozesse (z. B. Finanzen, Personal, Einkauf, interne Kommunikation) digital unterstützt. |

^a^Die Daten zu Items werden anhand einer 5-Punkte-Likert-Skala von 1 = „Trifft überhaupt nicht zu“ bis 5 = „Trifft voll und ganz zu“ erfasst.

^b^Die Daten zu den Items werden invertiert.
