# Supplementary figures and images for: Development and Validation of a Questionnaire to Measure Digital Maturity of General Practitioner Practices: Web-Based Cross-Sectional Survey Study
Source: J Med Internet Res. 2025 Oct 14;27:e81416. doi: 10.2196/81416 (PMC12569491; doi:10.2196/81416)

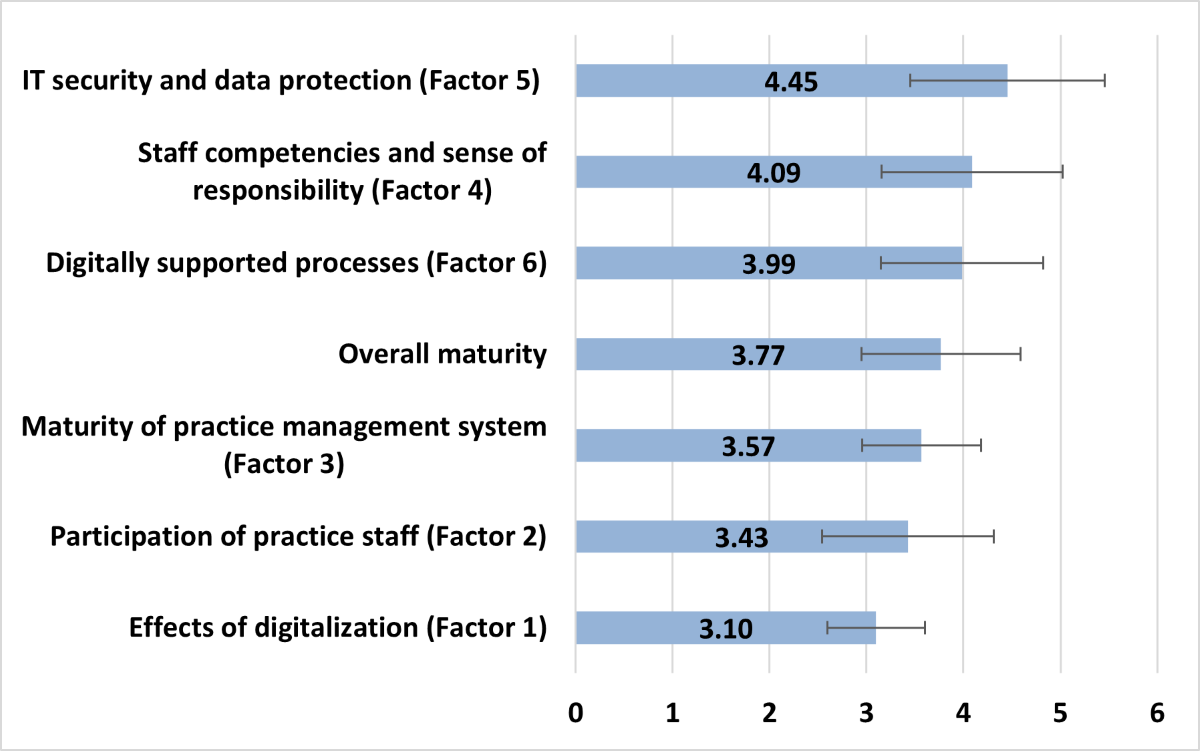

Supplement: Multimedia Appendix 5 [file jmir_v27i1e81416_app5.png]
